# A new strategy for the determination of the antidiabetics alogliptin, saxagliptin and vildagliptin using all-solid state potentiometric sensors

**DOI:** 10.1186/s13065-023-00988-1

**Published:** 2023-07-16

**Authors:** Abeer Rashad Derar, Neven Ahmed, Emad Mohamed Hussien

**Affiliations:** Egyptian Drug Authority (EDA), 9 Abou-Hazem str, P.O Box 29, Giza, Egypt

**Keywords:** Screen printed ion selective electrode, Au and Pt nanoparticles, All-solid-state electrode, Coated wire, Dipeptidyl peptidase-4 inhibitors

## Abstract

Herein, we report on the development of disposable screen printed carbon, nanostructure thin film Au/Pt and Pt/Pt all-solid state potentiometric sensors for some antidiabetic compounds called glibtins. The electrodes showed excellent calibration curves (1 × 10^–5^–1 × 10^–2^ M) for alogliptin, saxagliptin and vildagliptin. The electrodes were fully characterized with respect to potential stability, dynamic response time, detection limit, effect of pH and interference according to the IUPAC recommendation. The proposed method is rapid and can be applied for the determination of gliptins at low cost with satisfactory precision (RSD ≤ 1%) and accuracy.

**Supplementary Information:**

The online version contains supplementary material available at 10.1186/s13065-023-00988-1.

## Introduction

**Fig. 1:**
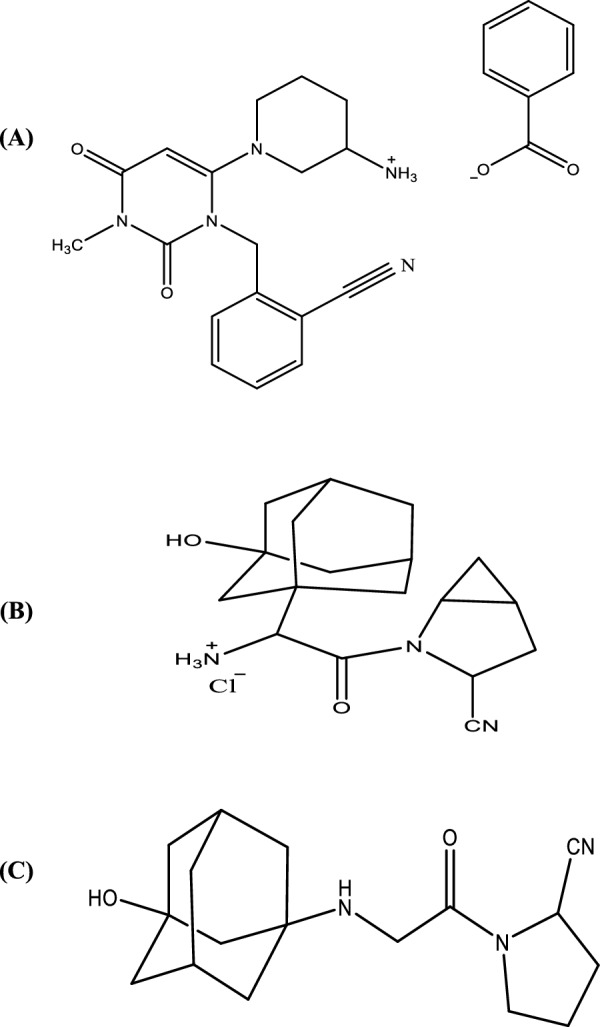
Molecular structures of **A** alogliptin benzoate, **B** saxagliptin hydrochloride and **C** vildagliptin

Alogliptin benzoate (ALO), saxagliptin hydrochloride (SAX) and vildagliptin (VIL) (Fig. 1) belong to a class of pharmaceutical compounds called gliptins which are prescribed for the treatment of type 2 diabetes [1]. The principal role of these compounds is to inhibit DPP-4 enzyme that destroys glucagon-like peptide-1 (GLP-1) and glucose-dependent insulin tropic peptide (GIP) hormones. These hormones are produced in the gut and are highly important for controlling blood glucose at normal levels by stimulating the secretion of glucagon and insulin in the pancreas. It has been reported that about 50% of the secreted GLP-1 is destroyed by DPP-4 before it reaches the general circulation and another 40% is destroyed before it reaches the pancreas [2]. Therefore, inhibiting DPP-4 is essential for diabetics. This critical pharmacological action has therefore been encouraging development of analytical methods to control gliptins in both pharmaceutical formulations and body fluids.

To this end, several HPLC and LC–MS/MS methods were developed for the assay of ALO [3–14], SAX [15–21] and VIL [5, 22–30]. Despite the fact that these techniques are superior in drug separation and analysis, they remain time and solvent consuming and use expensive instrumentations like (LC–MS/MS) that require, in most cases, outdoor analysis. Spectrophotometric and spectrofluorimetric methods have also been reported for ALO [31–34], SAX [35–37] and VIL [38–40]. These methods are based on derivative spectrophotometry with tedious calculations to circumvent convolutions. Additionally, the proposed spectrofluorimetric methods utilize highly reactive compounds for chemical derivatization which not only prolong the time scale of the analytical procedures but also require special safety measures and precautions.

Recently, a great attention has been focused on portable and printed electrochemical all-solid-state potentiometric sensors due to their simplicity, low cost and easy production in various sizes and designs [41–44]. Usually, solid-contact (SC) based ion—selective electrodes consist of a noble metal or graphite (printed or in the form of a rod) in direct contact with the sensing membrane (no inner filling solution) [45–48]. Long term potential stability of these electrodes has been achieved either by increasing the surface area of the solid contact or by using redox compounds at the membrane/solid contact interface [49–51]. For example, carbon nanotubes have been used for the fabrication of solid-contact K^+^ ion selective electrode (ISE) with high-performance and long-life [52, 53]. This Nanomaterial provides high surface area, high conductivity as well as the ability to function as an ion-to-electron transducer when used as an electron conductors in SC-ISEs [54]. Solid contact potentiometric ISEs with metal nanoparticles have also been reported for determination of K^+^ [45] and pharmaceutical compounds [55, 56].

In this work, we report for the first time the determination of ALO, SAX and VIL using stencil printed potentiometric strip fabricated at home from inexpensive material (graphite ink and a plastic sheet as a substrate). Moreover, coated wire ISEs with high surface area of the inner solid contact was constructed using miniaturized Pt wire with electrochemically deposited thin film of Au and Pt nanoparticles. The surface morphology of the Au/pt and Pt/Pt thin film was characterized using scanning electron microscopy (SEM) and the capacitance was evaluated using current reverse chronopotentiometry (CRC). Important potentiometric characteristics including linearity, potential stability, hysteresis and selectivity were evaluated according the IUPAC recommendations [57]. The proposed analytical strategy is aiming at the fabrication of highly stable potentiometric sensors that can be used for rapid and reliable determination of ALO, SAX and VIL at low cost.

## Experimental

### Material and reagents

Polyvinyl chloride (PVC) membrane components including PVC, dibutyl phthalate (DBP) and ortho nitrophenyl octyl ether (oNPOE) were obtained from Sigma-Aldrich, USA. Other components including tricresyl phosphate (TCP) and sodium tetraphenylborate (Na-TPB) belong to Fluka Company, Switzerland. Tetrahydrofuran and Lipohilic ion exchanger Potassium tetrakis(4-chlorophenyl)borate (KTCPB) were purchased from Alfa Aesar, Germany. Cyclohexanone was purchased from Sigma-Aldrich, USA and Acetone was from Thermo Fisher Scientific, USA. Hexachloroplatinic acid and graphite powder (particle size < 50 μm) were purchased from Merck, Germany. Tetrachloroaurate (III) hydrate was purchased from Alfa Aesar, Germany. Alogliptin Benzoate, saxagliptin HCl and vildagliptin drug substances (DS) were obtained from National Organization for Drug Control and Research (NODCAR), Giza, Egypt. All other solutions were prepared from analytical grade chemicals and double distilled water.

### Pharmaceutical formulations

Commercial pharmaceutical finished products were purchased from the local market and are as follows:

- Inhiglip (12.5 mg alogliptin benzoate/tablet) is produced by Hikm Pharma, Cairo, Egypt.
- Formigliptin (5 mg saxagliptin HCl/tablet) is manufactured by Multicare for Pharmaceutical Industries, Cairo, Egypt.
- Vildagluse (50 mg vildaglptin/tablet) is produced by Inspire Pharmaceutical Industries, Cairo, Egypt.

### Screen printed electrode

**Fig. 2:**
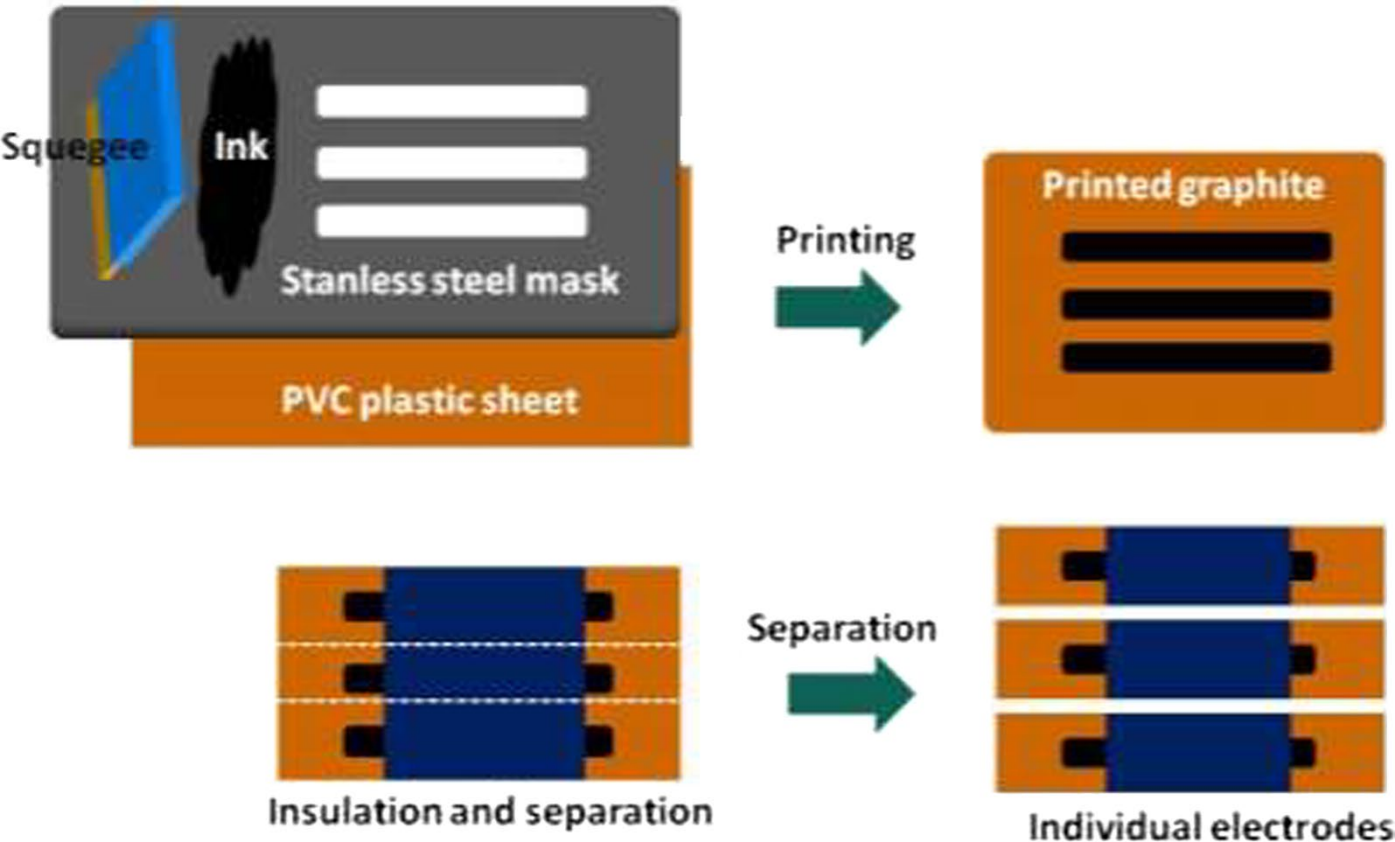
Illustration for the steps of preparation of stencil printed ion selective electrodes

Screen printed graphite electrodes were prepared using a home-made graphite ink which is by mixing 3 g PVC solution (8% in cyclohexanone–acetone mixture 1:1) and 1.5 g graphite powder. The ink was printed on a transparent PVC plastic sheet of 200 µm thick using a squegee (to help spread the ink) and custom-made stainless steel mask. The printed electrodes were left in a vacuum oven (Memmert, Germany) at 60 °C for 2 h. An insulating tape was placed over the electrode except areas of 3 × 3 mm at both ends of the electrode, one serves as inner solid contact for the ion selective electrode and the other is used to connect the electrode with potentiometer. Ion selective membrane (ISM) solution composed of 67.5% w/w plasticizer, 1.5% w/w KTCPB and 31.0% w/w PVC in 1:1 cyclohexanone: acetone (v/v) was drop casted onto the electrode surface and left to dry at room temperature for 24 h. Figure 2 shows the preparation of stencil printed ion selective electrodes using home-made graphite ink.

### Metal nanoparticles/Pt solid contact 

Pt solid contact electrdoe was prepared as described previously [55]. In brief, the electrode was cleaned using alumina paste of 15, 3 and 1 µm and then rinsed with deionized water. Pt nanostructures were electrochemically deposited onto the wire at − 1.0 V using 5 mM H_2_PtCl_6_ [45]. Onto another Pt electrode, porous Au thin nanostructured-film was formed by applying a potential of − 3.0 V in 5 mM HAuCl_4_ for 90 s [45]. The electrode was dipped several times in the membrane solution: 31.0 wt% PVC, 1.5 wt% KTCPB and 67.5 wt% oNPOE in THF. The solvent was allowed to evaporate at room temperature for 24 h.

### Standard solutions

Standard solutions of ALO^+^, SAX^+^ were prepared each in 50 mL measuring flask by dissolving 230.8 mg alogliptin benzoate and 194.0 mg saxagliptin hydrochloride dihydrate in 30 mL water, followed by completing the volume to the mark with water. VIL^+^ standard solution was prepared by dissolving an accurately weighed 151.7 mg of vildaglibtin in 50 mL of 0.1 M HCl.

### Potentiometric measurements

All potentiometric measurements were measured against Ag/AgCl (3 M KCl) reference electrode (BAS Inc, Japan). The measurements were carried out using Proskit MT-1820 digital multimeter operated by DMM data collector software or Jenway 3510 pH/mV meter (England). The potential was recorded for solutions of variable concentrations covering the range from 1 × 10^–6^ up to 1 × 10^–2^ M of the target cation (ALO^+^, SAX^+^ or VIL^+^). Calibration curves were constructed by plotting the potential reading (mV) *vs.* -log [conc., M]. The influence of pH on the potentiometric response was examined using 1 × 10^–3^ M of the target ion and changing the pH of the solution using HCl and/or NaOH solutions and recording the variation in the electrode potential.

### Selectivity

The selectivity of the electrode was studied using fixed interference method [58]. The equilibrium potential was measured for solutions of constant concentration (10^–2^ M) of the interfering ion, *a*B, and varying concentrationy of the drug ion, *a*A. This was done by adding small aliquets from 10^–2^ M solutions of the drug ion to 50 mL of 10^–2^ M of the interfering ion. Then, the potential values were recorded and plotted vs. the logarithm of the concentration of the drug ion. The two linear segments of the plot were extrapolated and the value of *a*A at the intersection was used to calculate the selectivity coefficient ($${K}_{A,B}^{Pot.})$$ from Eq. 1:

1 $$K_{A,B}^{Pot} = a_{A} /(a_{B} )^{{{\raise0.7ex\hbox{${z_{A} }$} \!\mathord{\left/ {\vphantom {{z_{A} } {z_{B} }}}\right.\kern-0pt} \!\lower0.7ex\hbox{${z_{B} }$}}}}$$

where *z*A and *z*B are charge numbers of the primary ion (drug), A, and of the interfering ion, B

### Analytical applications

- **Sample solutions**
  **ALO**^+^** sample solution (0.01 M).** Thirty tablets of Inhiglip tablets were finely ground in a mortar and a small amount containing 230.8 mg aloglibtine benzoate (based on the label claim) was accurately weighed and transferred into 50 mL volumetric flask, dissolved in 30 mL double distilled water, sonicated for 10 min and the volume was completed to mark with water.
  **SAX**^+^** sample solution (0.01 M).** Sixty tablets of Formigliptin were finely ground in a mortar and an amount of the powder equivalent to 194.0 mg Saxaglibtine hydrochloride was accurately weighed and transferred into 50 mL volumetric flask. The final solution was prepared as above.
  **VIL**^+^** sample solution (0.01 M)**. Ten tablets of Vildagluse were finely ground in a mortar and an amount of the powder equivalent to 151.7 mg vildaglibtin was accurately weighed and transferred into 50 mL volumetric flask. The powder was dissolved in 30 mL 0.1 N HCl, sonicated for 10 min., then, the flask was completed to the mark with 0.1 N HCl.
- **Standard addition method**
  In this method, known small volumes (${\mathrm{V}}_{\mathrm{s}}$) of standard solution of 0.01 M of the target analyte is added to 50 mL of the sample solution. The change in the electrode potential (mV) was recorded after each addition and used to calculate the concentration of the drug in the sample solution using Eq. 2:
  2 $${\text{C}}_{{\text{x}}} = {\text{C}}_{{\text{s}}} \left( {\frac{{{\text{V}}_{{\text{s}}} }}{{{\text{V}}_{{\text{x}}} + {\text{V}}_{{\text{s}}} }}} \right)\left[ {10^{{\Delta {\text{E}}/{\text{S}}}} - \left( {\frac{{{\text{V}}_{{\text{x}}} }}{{{\text{V}}_{{\text{x}}} + {\text{V}}_{{\text{s}}} }}} \right)} \right]^{{ - 1}}$$
  Where: Cx: unknown concentration of the sample, Vx: volume of the sample, Cs and Vs: concentration and volume of the standard solution, ∆E: change in mV reading of the sample solution due to addition of standard solution and S is the slope of the electrode.

## Results and discussion

### Potentiometric characteristics

**Fig. 3:**
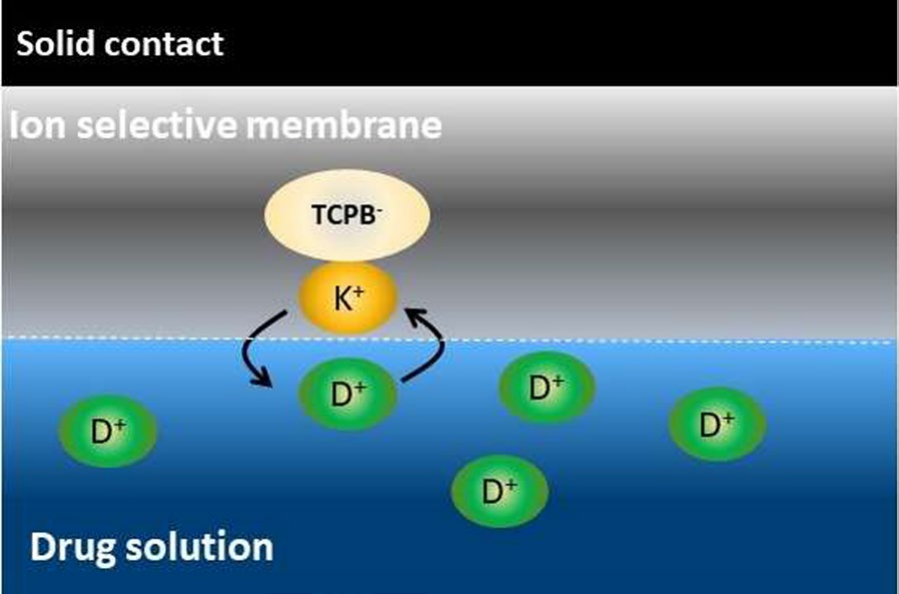
Schematic representing the replacement of K^+^ with the drug cation (D^+^) from the solution and formation of D-TCPB ion exchanger in the membrane phase. D^+^ stands here for ALO^+^, SAX^+^ and VIL^+^. When the membrane comes in contact with the drug solution an ion exchange process is established between D^+^ in the membrane and that in the solution, and D^+^ becomes potential determining ion

**Fig. 4:**
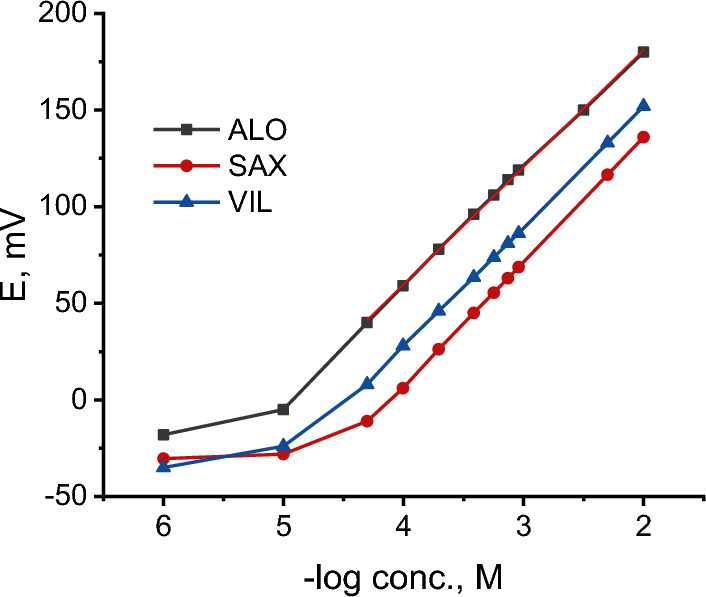
Calibration curves for ALO^+^, SAX^+^ and VIL^+^ using KTCPB as ion exchanger and DBP as plasticizer

**Fig. 5:**
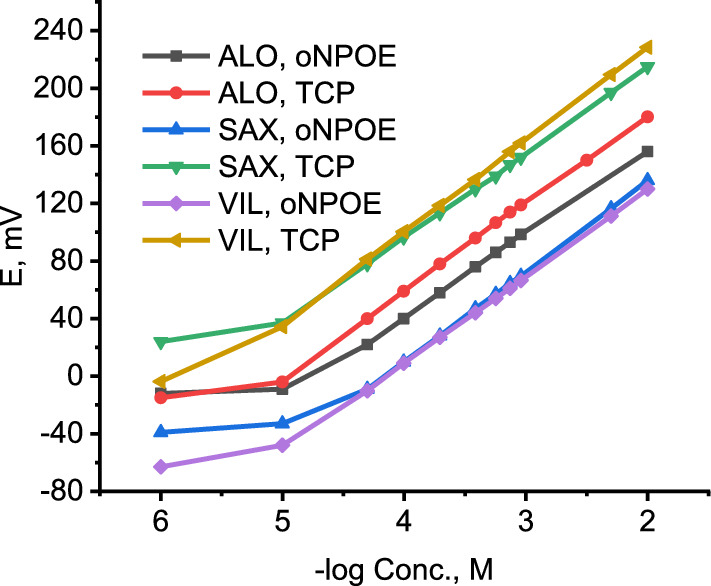
Calibration curves for ALO^+^, SAX^+^ and VIL^+^ using oNPOE and TCP plasticizers

**Fig. 6:**
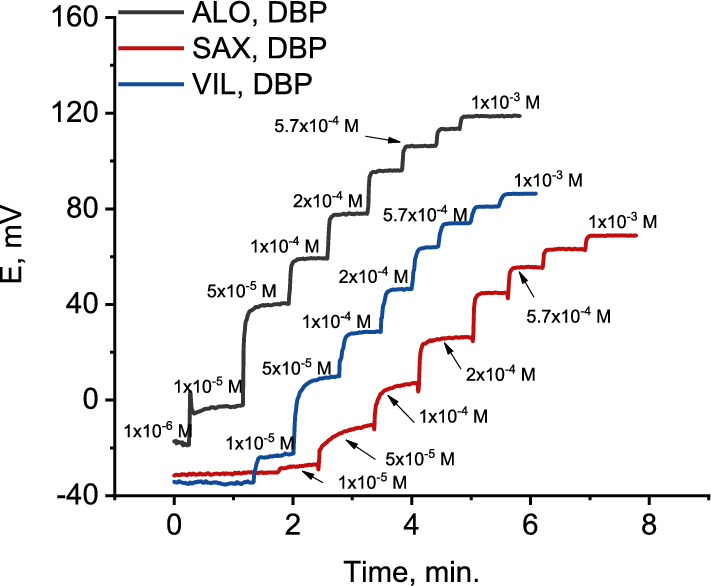
Potentiometric response (ΔE vs. t) of the electrode over the concentration range from 1 × 10–6 to 1 × 10–3 M for ALO + , SAX + and VIL +

- a. Selection of the ion exchanger
  The sensing element is the most important component in potentiometric ion selective electrode and should be of high lipophilicity, soluble in the membrane matrix and chemically stable under the experimental conditions [59]. Such characteristics are essential for ion selective electrodes with long life time and lower detection limits. To this end, several ion exchangers were formed in aqueous solution by co-precipitation of the target cation with phosphomolybdic acid, ammonium reineckate or sodium tetraphenylborate. However, all of them were found to be soluble in water and, hence, were useless for ISE preparation.
  Conversely, incorporation of highly lipophilic KTCPB (practically insoluble in water) in a membrane matrix containing DBP and conditioning the electrode in the target analyte solution resulted in an *in-situ* ion exchanger (ALO-TCPB, SAX-TCPB and VIL-TCPB) formation in the membrane phase (Fig. 3). Nernestian responses of 60.6, 63.0 and 62.0 mV/decade were obtained for ALO^+^, SAX^+^ and VIL^+^, respectively, after conditioning the electrode in the target analyte for 30 min. the potentiometric characteristics of *in-situ* ion exchanger based electrode for ALO^+^, SAX^+^ and VIL^+^ are summarized in Table 1; and the corresponding calibration curves are shown in Fig. 4. These results refer to a fast ion exchange process where the drug cation from the conditioning solution replaces the counter ion (K^+^) in the membrane matrix.
- b. Effect of plasticizes
  In addition to DBP, the influence oNPOE and TCP plasticizers on the potentiometric characteristics of the electrode was studied. Typical calibration curves for ALO^+^, SAX^+^ and VIL^+^ are shown in Fig. 5. oNPOE based electrode showed Nernstian slopes of 58.5, 62.8 and 60.4 mV/decade for ALO^+^, SAX^+^ and VIL^+^, respectively. Meanwhile, TCP based electrode showed Nernstian responses of 60.7 mV/decade, 59.2 and 64.4 mV/decade for ALO, SAX^+^ and VIL^+^, respectively. Obviously, the investigated plasticizers have no significant effect on the potentiometric characteristics of developed electrodes. The potentiometric response characteristics including the slopes, linear ranges and detection limits for ALO^+^, SAX^+^ and VIL^+^ using different plasticizers are summarized in Table 1.
- c. Dynamic response time
  The time that passes between the instant of touching the electrode to the sample solution and the instant at which the potential reading becomes stable and approaching a limiting value (ΔEΔ/t) is defined as the response time [57]. Figure 6 shows dynamic potentiometric responses recorded for stepwise increase in the concentration from 1 × 10^–6^ to 1 × 10^–3^ M for ALO^+^, SAX^+^ and VIL^+^, respectively, using DBP based electrode. A fast response time was reached in ≤ 20 s. However, the limiting value (ΔE/Δt) was in the order 1 × 10^–3^ < 1 × 10^–4^ < 1 × 10^–5^ M for all gliptins, with ΔE/Δt ≤ 0.11 mV/min in 1 × 10^–3^ M. Comparable results were obtained using oNPOE and TCP plasticizers for ALO^+^, SAX^+^ and VIL^+^ (Additional file 1: Fig S1). The obtained fast response time and small potential drift of the electrodes guarantee high accuracy for analytical applications.

**Table 1:** Potentiometric response characteristics for ALO^+^, SAX^+^ and VIL^+^ ion selective electrodes

Plasticizer	Slope (mV/decade)^a^	Linearity (M)	DL (M)	r^2^
ALO^+^				
DBP	60.6 ± 1	1 × 10^–5^–1 × 10^–2^	1.0 × 10^–5^	0.9995
oNPOE	58.5 ± 1	1 × 10^–4^–1 × 10^–2^	1.4 × 10^–5^	0.9990
TCP	57.7 ± 1	1 × 10^–4^–1 × 10^–2^	1.7 × 10^–5^	0.9994
SAX^+^				
DBP	62.0 ± 1	5 × 10^–5^–1 × 10^–2^	2.8 × 10^–5^	0.9999
oNPOE	62.8 ± 1	5 × 10^–5^–1 × 10^–2^	2.8 × 10^–5^	0.9999
TCP	59.2 ± 1	1 × 10^–5^–1 × 10^–2^	1.0 × 10^–5^	0.9997
VIL^+^				
DBP	62.0 ± 1	5 × 10^–5^–1 × 10^–2^	2.8 × 10^–5^	0.9998
oNPOE	60.4 ± 1	1 × 10^–5^–1 × 10^–2^	1.0 × 10^–5^	0.9999
TCP	64.4 ± 1	1 × 10^–5^–1 × 10^–2^	2.2 × 10^–6^	0.9998

^a^Average of three determinations

### Hysteresis (electrode memory)

**Fig. 7:**
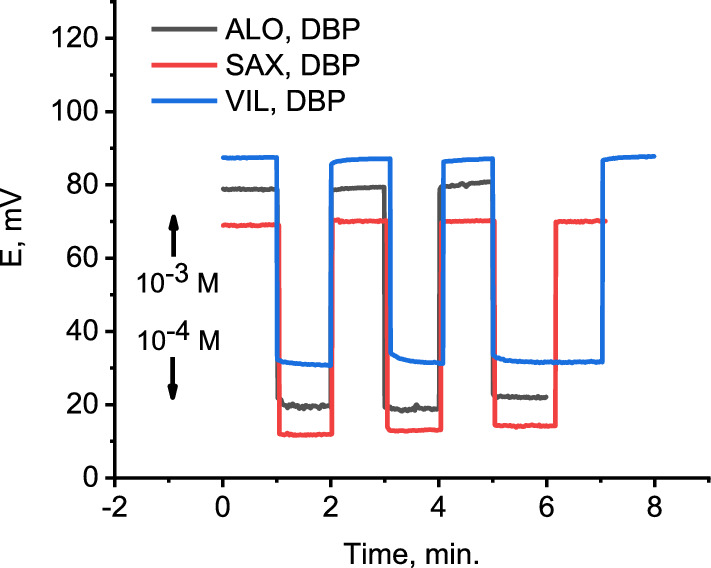
Potential reproducibility of DBP based electrode when it is alternatively immersed in 1 × 10^–4^ and 1 × 10^–3^ M of ALO^+^, SAX^+^ and VIL^+^. The potentiometric reproducibility curve of ALO^+^ is displaced for clarity

Hysteresis of ISEs has been defined by the IUPAC [57] as the inability of the electrode to recover its potential reading in a definite concentration of the analyte (solution A). Afterword, it is subjected to a solution containing a discriminated of less preferred ion (solution B). The difference between the two readings in solution A (before and after the electrode is exposed to solution B) is called hysteresis. Herein, the hysteresis was investigated by alternatively recording the electrode potential of DBP based electrode in 1 × 10–3 M and 1 × 10–4 M of ALO+, SAX+ and VIL+ (Fig. 7). Obviously, the potential of the electrode dropped immediately and jumped quickly to almost the same potential value (SD ≤  ± 1 mV, n = 3) in 1 × 10–3 M and 1 × 10–4 M, indicating a negligible memory effect. The hysteresis oNPOE and TCP based electrodes are shown in Additional file 1: Fig S2.

### Potential drift 

**Fig. 8:**
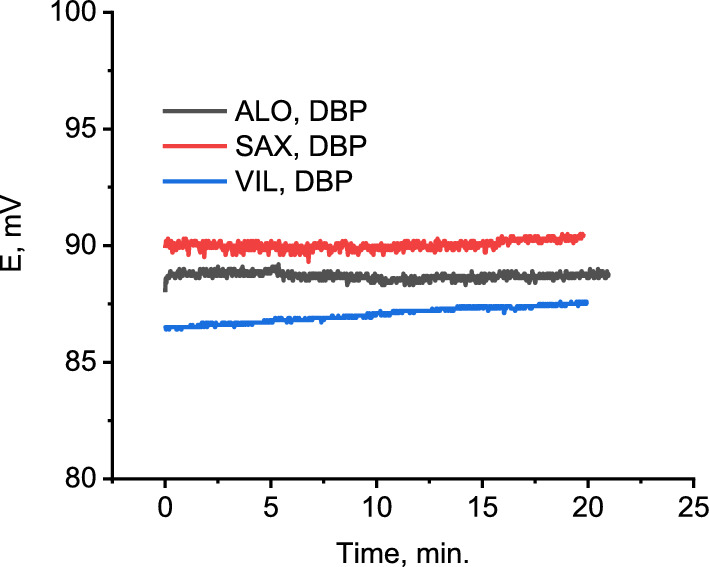
Potential stability of DBP based electrode monitored for about 20 min in 1 × 10^–3^ M of ALO^+^, SAX^+^ and VIL^+^. Potential stability curves of ALO^+^ and SAX^+^ are displaced for clarity

The potential drift (ΔE/Δt) is the change that is observed in potential when the electrode is immersed in the analyte solution for a period of time [57]. It would be due to degradation of the membrane components or polarization of the ion selective membrane/solid contact (ISM/SC) interface. In this work, the potential drift of the printed ISE was monitored over 20 min in a stirred solution of 10^–3^ M of the drug. The potential drift was found to be 0.16, 0.27 and 0.55 µV/s for ALO^+^, SAX^+^ and VIL^+^, respectively, using DBP based electrode (Fig. 8). The small potential drift over 20 min refers to the high potential stability and less polarization of ISM/SC interface. The potential drift recorded for oNPOE and TCP based electrodes is shown in Additional file 1: Fig S3.

### Effect of the internal solid contact

**Fig. 9:**
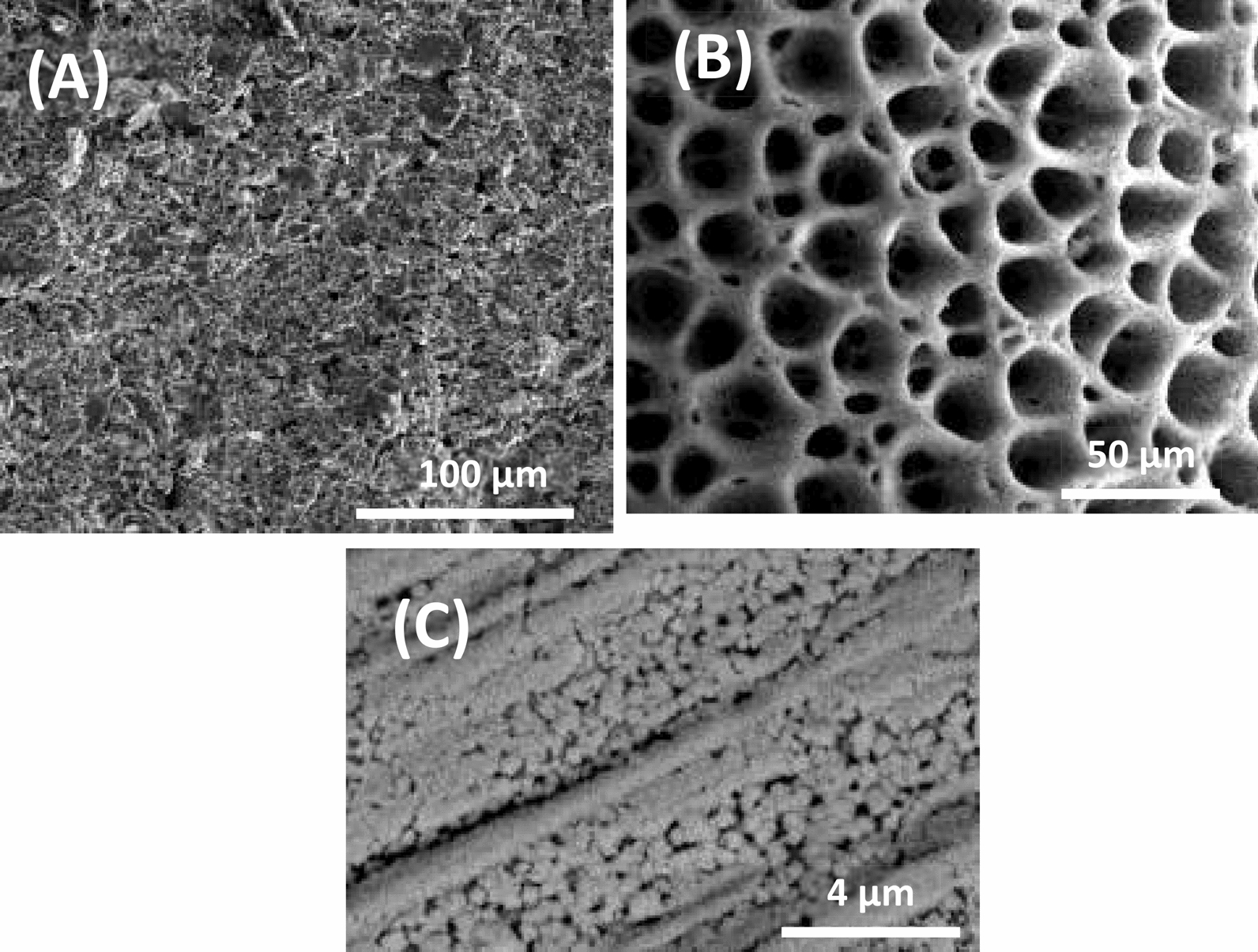
SEM pictures of stencil printed graphite (**A**), Au spongy film electrochemically deposited onto a pt wire (**B**) and electrochemically deposited Pt onto a Pt wire (**C**)

**Fig. 10:**
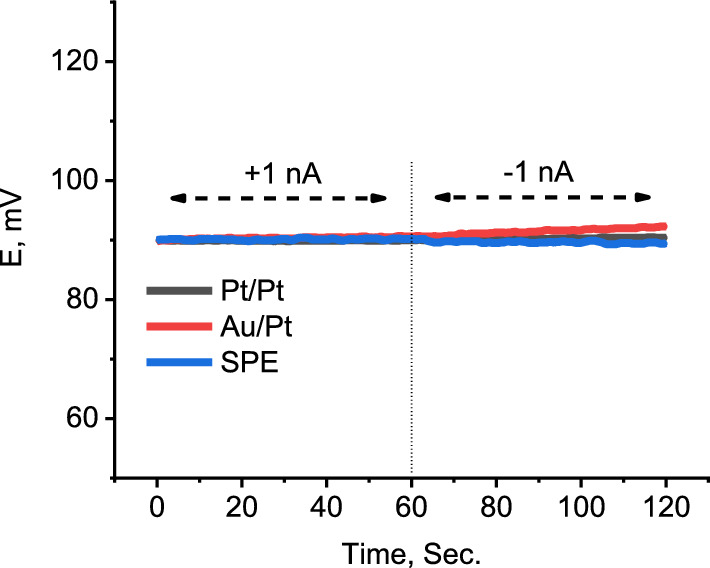
Chronopotentiogram of DBP based electrode with different internal solid contacts: Screen printed carbon, Au nanostructure thin film/Pt and Pt/Pt wire. The measurements were performed in 1 × 10^–3^ M ALO^+^. The applied current is + 1 nA and − 1 nA for 1 min

Current reverse chronopotentiometry (CRC) was used to evaluate the capacitance of the ISM/SC interface [60]. Although the potentiometric measurements are carried out with a high impedance device with extremely residual current (≤ pA), ISEs with large capacitance is essential for potential stability and accurate results which are relevant to the actual concentration. Various inner solid contact including printed graphite, Au and Pt nanostructures electrochemically deposited onto a Pt wire were employed. SEM pictures of the SPE, Au/pt and Pt/Pt inner solid contacts are shown in Fig. 9. The SEM picture of the SPE showed an irregular porous surface with well-connected carbon flakes, indicating a high surface area of the printed graphite. The picture also shows that Au/Pt surface is highly porous spongy-like surface which significantly affects the surface area of the electrode. The capacitance of the interface was evaluated by immersing the electrode in 10–3 M ALO and a current of ± 1 nA was applied for 60 s while monitoring the electrode potential (Fig. 10). The capacitance of the interface ($C=i\times \Delta t/\Delta E$ [60]) was calculated and was found to be 500, 142 and 1660 µF for SPE, Au/Pt and Pt/Pt electrodes, respectively. The high capacitance of the Pt/Pt is most probably due to the combination of the high surface area provided by nanostructure Pt film and the presence of a redox couple at the interface.

### Effect of pH

**Fig. 11:**
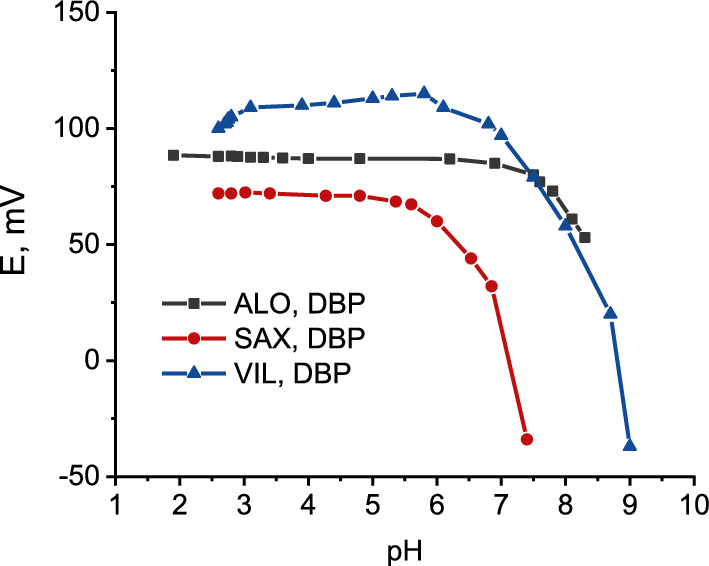
Effect of pH on the potential stability of DBP screen printed electrode in 1 × 10^–3^ M of ALO^+^, SAX^+^ and VIL^+^

The mV response curves as a function change in pH for ALO^+^, SAX^+^ and VIL^+^ using DBP based electrode are shown in Fig. 11. It is clear that the electrode potential is stable in the pH range from 2 to 6.9, 2.5 to 5.4 and from 2.5 to 5.8 for ALO^+^, SAX^+^ and VIL^+^, respectively. The dramatic response observed at higher pH values would be due to the conversion of the drug from the ionized form to the base form (unprotonated species). The effect of pH on the TCP based electrode for ALO^+^, SAX^+^ and VIL^+^ is shown in Additional file 1: Fig S4.

### Effect of interference

The interference from common interfering compounds was studied by the fixed interference method. The interference is expressed by the selectivity coefficient ${k}_{A,B}^{pot}$ where high value of ${k}_{A,B}^{pot}$ refers to a high interference. Because the selectivity of the developed electrode for the interfering compounds was very small compared to the analyte, the selectivity coefficients are reported as logarithm values in Table 2. The reported values refer to negligible interference from the studied compounds.

**Table 2:** Potentiometric selectivity coefficients of the SPE

		$$log{k}_{A,B}^{pot}$$	
Interferant	ALO^+^	SAX^+^	VIL^+^
K^+^	− 2.98	− 2.61	− 2.98
Na^+^	− 3.02	− 2.45	− 2.90
NH^4+^	− 3.06	− 2.48	− 2.82
Ca^2+^	− 3.96	− 3.39	− 2.97
Mg^2+^	− 3.89	− 3.55	− 3.03
Leucine	− 3.14	− 2.27	− 2.76
Glycine	− 3.22	− 2.38	− 2.71
Glutamine	− 3.19	− 2.23	− 2.68
Alanine	− 3.09	− 2.19	− 2.60
Lysine	− 3.17	− 2.28	− 2.65

## Analytical applications

The proposed electrodes (DBP, oNPOE and TCP based electrodes) exhibited comparable Nernestian responses, fast response time, satisfactory reproducibility and high selectivity. However, the DBP based electrode was selected for the determination of ALO^+^, SAX^+^ and VIL^+^ in the pharmaceutical tablets because it showed the lowest potential drift for ALO^+^ (0.16 µV/s), SAX^+^ (0.27 µV/s) and VIL^+^ (0.93 µV/s). The accuracy and precision were evaluated using the standard addition method. The average recovery (n = 3) was found to be > 97.0% and < 102.0% referring to high accuracy of determination of ALO^+^, SAX^+^ and VIL^+^. The %RSD (n = 9) was found to be ≤ 1% indicating a satisfactory precision of the method. The accuracy and precision results for the drug substances (DS) and pharmaceutical formulations are summarized in Table 3.

**Table 3:** Accuracy and precision of the method for the determination of ALO^+^, SAX^+^ and VIL^+^ in the drug substances and pharmaceutical formulations

Drug		Conc. (mg/mL)	Found (mg/mL)	± SD	%Recovery (n = 3)	%Recovery (n = 9)	%RSD
ALO^+^		0.0462	0.0455	0.0005	98.50		
	DS	0.0923	0.0909	0.0016	98.53		
		0.1385	0.1346	0.0017	97.21		
		0.0462	0.0455	0.0003	98.58		
	Tablet	0.0923	0.0911	0.0015	98.72	98.31	0.60
		0.1385	0.1352	0.0016	97.63		
SAX^+^							
		0.0388	0.0380	0.0004	97.88		
	DS	0.0776	0.0760	0.0008	98.02		
		0.1164	0.1147	0.0023	98.56		
		0.0388	0.0376	0.0005	97.04		
	Tablet	0.0776	0.0758	0.0008	97.73	97.70	0.67
		0.1164	0.1144	0.0018	98.34		
VIL^+^							
		0.0303	0.0310	0.0005	102.16		
	DS	0.0607	0.0607	0.0006	100.03		
		0.0910	0.0924	0.0010	101.55		
		0.0303	0.0305	0.0007	100.54		
	Tablet	0.0607	0.0599	0.0005	98.71	99.61	0.92
		0.0910	0.0906	0.0010	99.58		

The accuracy results of determination of ALO^+^, SAX^+^ and VIL^+^ obtained by the present electrode were compared with those obtained by published methods using the student t-test.

The calculated t-values for six replicates (n = 6) were found to be 1.86, 2.05 and 2.35 for ALO, SAX and VIL, respectively. These values are less than the tabulated value of t at P = 0.05 indicating that the present method is equivalent to the published methods with regard to the accuracy.

Moreover, the precision of our method was compared with those obtained by the published methods using the F ratio test. This test is used to compare the standard deviations (random error) of two sets of data. The obtained F values for six replicates were 1.14, 1.19 and 1.38 for ALO, SAX and VIL, respectively. Obviously, the F values are less that the tabulated value at P = 0.05, indicating that the present method is equivalent to the published methods in terms of method precision. The calculated student t-test and F-ratio values are summarized in Table 4. Additionally, the proposed method has several advantages over the reported HPLC methods [14, 18, 26] including the measurements in turbid solutions, rapid measurements and use no hazardous organic solvent.

**Table 4:** Statistical comparison between the proposed ISE method and reported method for the determination of ALO^+^, SAX^+^ and VIL^+^ in pharmaceutical dosage forms

	Parameter	Present method	Published method [14]
ALO^+^	% Mean recovery	98.3	100.1
	SD	1.56	1.78
	Variance	2.43	3.17
	n	6	6
	T-test (2.36)^a^	1.86	
	F-test (5.05)^a^	1.14	
			Published method [18]
SAX^+^	% Mean recovery	98.8	100.4
	SD	1.23	1.46
	Variance	1.51	2.13
	n	6	6
	T-test (2.36)^a^	2.05	
	F-test (5.05)^a^	1.19	
			Published method [26]
VIL^+^	% Mean recovery	97.8	99.4
	SD	0.98	1.35
	Variance	0.96	1.82
	n	6	6
	T-test (2.36)^a^	2.35	
	F-test (5.05)^a^	1.38	

[^a^] Values between parentheses are the tabulated *t-* and F values, at P = 0.05

## Conclusion

The present work describes the fabrication and characterization of portable all-solid-state potentiometric sensors for determination of dipeptidyl peptidase-4 inhibitors (DPP-4 s) including alogliptine benzoate, saxagliptin HCl and vildgliptin. The screen printed sensor was constructed by printing PVC membrane containing KTCPB onto disposable graphite stripes. Miniaturized portable Pt electrodes with high surface area were prepared by no-manual deposition of Pt and Au nanostructures. The potentiometric response to ALO^+^, SAX^+^ and VIL^+^ was found to be Nernstian with slopes NLT 58.5 mV/decade and NMT 64.5 mV/decade. A wide linear response from 1 × 10^–5^ to 1 × 10^–2^ M was observed for ALO^+^, SAX^+^ and VIL^+^. The sensors showed high reproducibility, fast response time and potential stability as it was revealed by CRC. The proposed sensors exhibited high selectivity and have been applied successfully for rapid determination of ALO^+^, SAX^+^ and VIL^+^ in their pharmaceutical formulations.

## Supplementary Information

**Additional file 1: Figure S1.** Dynamic response time (ΔE vs. t) covering the concentration range from 1 x10-6 to 1 x10-3 M for ALO+, SAX+ and VIL+. Some curves are displaced for clarity. **Figure S2.** Potential reproducibility of SPEs based on oNPOE and TCP as plasticizers. The electrode is alternatively immersed in 1 x10-4 and 1 x10-3 M of ALO+, SAX+ and VIL+. while monitoring the potential change. **Figure S3.** Potential stability of SPEs based on oNPOE and TCP as plasticizers. The potential is monitored in 1x10-3 M of ALO+, SAX+ and VIL+. for about 20 min. Some curves are displaced for clarity. **Figure S4.** Effect of pH on the potential stability of TCP screen printed electrode in 1x10-3 M of ALO+, SAX+ and VIL+.

## Data Availability

All raw data and materials on which this study is based are included in the manuscript.
