# Supplementary material for: A new strategy for the determination of the antidiabetics alogliptin, saxagliptin and vildagliptin using all-solid state potentiometric sensors
Source: BMC Chem. 2023 Jul 16;17(1):79. doi: 10.1186/s13065-023-00988-1 (PMC10350275; doi:10.1186/s13065-023-00988-1)
Supplement: Supplementary file 1 — Additional file 1: Figure S1. Dynamic response time (ΔE vs. t) covering the concentration range from 1 x10^-6^ to 1 x10^-3^ M for ALO+, SAX+ and VIL+. Some curves are displaced for clarity. Figure S2. Potential reproducibility of SPEs based on oNPOE and TCP as plasticizers. The electrode is alternatively immersed in 1 x10^-4^ and 1 x10^-3^ M of ALO+, SAX+ and VIL+. while monitoring the potential change. Figure S3. Potential stability of SPEs based on oNPOE and TCP as plasticizers. The potential is monitored in 1x10^-3^ M of ALO+, SAX+ and VIL+. for about 20 min. Some curves are displaced for clarity. Figure S4. Effect of pH on the potential stability of TCP screen printed electrode in 1x10^-3^ M of ALO+, SAX+ and VIL+. [file 13065_2023_988_MOESM1_ESM.docx]

***Additional file 1***

**A New strategy for the determination of the antidiabetics Alogliptin, Saxagliptin and Vildagliptin using All-solid state potentiometric sensors**

**Abeer Rashad Derar, Neven Ahmed and Emad Mohamed Hussien^*^**

Egyptian Drug authority (EDA), Giza, Egypt 9 Abou-Hazem str, Giza, Egypt.

*Correspondence author at: Egyptian Drug authority, P.O Box 29, Giza, Egypt. Tel.: +2 02 3749 6077.

*E-mail address:* emadhussien@yahoo.com (Emad M. Hussien)

Figure S1. Dynamic response time (ΔE *vs.* *t*) covering the concentration range from 1 x10^-6^ to 1 x10^-3^ M for ALO^+^, SAX^+^ and VIL^+^. Some curves are displaced for clarity.

Figure S2. Potential reproducibility of SPEs based on oNPOE and TCP as plasticizers. The electrode is alternatively immersed in 1 x10^-4^ and 1 x10^-3^ M of ALO^+^, SAX^+^ and VIL^+^. while monitoring the potential change.

Figure S3. Potential stability of SPEs based on oNPOE and TCP as plasticizers. The potential is monitored in 1x10^-3^ M of ALO^+^, SAX^+^ and VIL^+^. for about 20 min. Some curves are displaced for clarity.

Figure S4. Effect of pH on the potential stability of TCP screen printed electrode in 1x10^-3^ M of ALO^+^, SAX^+^ and VIL^+^..
